# Effect of a Standardized Four-Week Desensitization and Counter-Conditioning Training Program on Pre-Existing Veterinary Fear in Companion Dogs

**DOI:** 10.3390/ani9100767

**Published:** 2019-10-07

**Authors:** Anastasia Stellato, Sarah Jajou, Cate E. Dewey, Tina M. Widowski, Lee Niel

**Affiliations:** 1 Department of Population Medicine, Ontario Veterinary College, University of Guelph, Guelph, ON N1G 2W1, Canada; astellat@uoguelph.ca (A.S.); sjajou@uoguelph.ca (S.J.); cdewey@uoguelph.ca (C.E.D.); 2 Department of Animal Biosciences, Ontario Agricultural College, University of Guelph, Guelph, ON N1G 2W1, Canada; twidowsk@uoguelph.ca

**Keywords:** dogs, training, counter-conditioning, desensitization, fear, veterinary clinic, behaviour, welfare

## Abstract

**Simple Summary:**

Reducing veterinary fear in dogs is important for canine health and welfare. It is commonly suggested that dog owners perform counter-conditioning and desensitization training to reduce veterinary fear levels, yet the efficacy of this training has not been evaluated. We recruited owned dogs with pre-existing fear and conducted mock veterinary appointments before and after training to assess changes in fear responses. Owners of dogs in the training group (n = 15) were instructed to perform exam-style handling and to visit the veterinary clinic on a weekly basis for four weeks, and owners of control dogs (n = 22) received no instructions. Compliance to training protocols was poor, with 44% of owners non-compliant to the training program. We found that, during the examination, trained dogs had less reduced posture than control dogs, but trained dogs showed more lip licking during clinic entrance and examination. Owners reported an observable improvement in their dog’s fear levels across the training sessions, and general fear scores lowered during the second examination for trained dogs. We suggest that, although few behavioural indicators of fear changed in the predicted direction, veterinarians should continue to recommend this training until further research is conducted.

**Abstract:**

Many dogs show signs of fear during veterinary appointments. It is widely recommended to use desensitization and counter-conditioning training to reduce this fear. However, the efficacy of this method for reducing veterinary fear has not been examined. We assessed the effect of a standardized four-week training program on behavioural and physiological signs of fear in dogs with pre-existing veterinary fear. Owned dogs were randomly allocated to receive training (n = 15) or no training (n = 22; Control). Owners of dogs in the training group were instructed to perform exam-style handling on their dog and to visit the veterinary clinic weekly. Owners of control dogs were given no instructions. Fear responses were assessed before and after the training period by a blinded observer during clinic arrival and examination. Despite motivated owners volunteering to participate in the current study, 44% of owners were non-compliant to this training program. During examination, control dogs had higher odds (95% confidence Interval (CI)) of reduced posture compared to trained dogs (Odds ratio (OR): 3.79, CI: 1.03–16.3). Fear scores for trained dogs lowered during the second examination (*p* = 0.046), and 86.7% of dog owners reported a reduction in their dog’s fear levels across the training period (*p* = 0.007). When entering the clinic (*p* = 0.002) and during examination (*p* = 0.002), trained female dogs had a higher rate of lip licking than control females. The training program did not influence temperature, heart rate, respiratory rate, avoidance, trembling, vocalizations, or willingness and encouragement to step on the scale. Results suggest that this four-week training program was mildly effective at reducing veterinary fear in dogs. Further research is necessary to explore the efficacy of longer, more intensive, and individualized training programs.

## 1. Introduction

Recent reports suggest that routine veterinary visits cause stress and fear in many companion dogs. For example, one study found that over 50% of dogs showed signs of fear while waiting in the reception area [1], while another study found that 78.5% of dogs were categorized as “fearful” across various components of a veterinary exam [2]. This fear may be a contributor to a recently reported decline in veterinary attendance for both dog and cat owners in the USA [3]. When asked about factors influencing the frequency of veterinary visits, 22% of dog owners reported they would visit the veterinarian more often if their dog was less stressed [3]. This veterinary-related fear can also reduce the ability for veterinary staff to conduct thorough examinations [4] and can lead to inaccurate diagnostic test results [5,6]. In addition, when fear progresses to aggression, it can pose a safety risk for veterinarians. One study found that 16% of dogs were aggressive during a veterinary examination [4], and a survey of veterinarians from Minnesota and Wisconsin found that 92.3% of participants reported at least one previous in-clinic dog bite [7]. Thus, prevention and treatment strategies are needed to reduce veterinary-related fear and aggression in dogs.

Dog fear in the clinic and during physical examinations can be persistent, even when improvements are made to the environment and to handling to reduce dog fear levels. For example, dog fear in veterinary settings is reduced when background noise is minimized [8] and when owners are present and interact with dogs during their examinations [9,10,11], but dogs still display signs of fear even with these alterations. Thus, strategies are needed to prevent and treat dog fear towards the veterinary environment and the physical examination. Immediate strategies that are recommended for use in clinics include using minimal restraint during handling [12,13,14,15], performing the examination where the animal is most comfortable [15], using chemical restraint [12,13,16], and providing treats and distraction to make experiences more positive [12,15,17]. However, appointments are infrequent and of limited duration, and these types of adjustments are unlikely to result in major improvements in dogs with pre-existing fear. Therefore, effective treatment programs are needed for reducing fear responses to relevant veterinary stimuli and experiences to improve responses between appointments.

Systematic desensitization and counter-conditioning are training methods that can be used to change emotional responses associated with particular stimuli [17]. Systematic desensitization involves repeated, gradual exposure to the stimulus that evokes a negative emotional response, and counter-conditioning involves pairing of the stimulus with something favourable (e.g., treats) to alter the emotional response. Counter-conditioning has shown to be effective in reducing fear across a variety of species, such as humans [18], rats [19], and horses [20]. For example, humans showed less avoidance of spiders [18], rats were more willing to approach their conditioned feared stimulus [19], and horses had a reduced heart rate and fewer flight responses when interacting with a moving nylon bag [20]. In companion dogs, desensitization and counter-conditioning has been effective at reducing aggression towards dogs and unfamiliar people [21,22] and fireworks-induced fear [23,24]. Although this training strategy is widely recommended for reducing fear in veterinary settings, the efficacy of a specific desensitization and counter-conditioning protocol in reducing fear associated with veterinary care has yet to be assessed.

Many stimuli have been suggested to contribute to canine fear and might influence responses during veterinary care, including novel environmental features [25], unfamiliar humans [26], unfamiliar handling [27,28], loud noises [29], and pain or discomfort experienced during routine procedures experienced, such as vaccinations [12]. Since many of these stressful exposures and painful procedures begin occurring in early life, there is the potential for this response to be exacerbated over time and become more severe with repeated exposure. In order to effectively change the dog’s emotional response towards the clinic environment, desensitization and counter-conditioning programs need to target these particular stimuli. It has been suggested that this training practice is most effective when the unconditioned stimulus (e.g., treats) is offered when the dog is below their fear threshold [30]. It is impractical to perform this type of training during routine appointments, thus a separate owner-completed program is an alternative approach that can be applied outside of regular appointment times. If this type of training is effective, it would reduce fear levels during clinic visits and facilitate future visits, which would benefit the welfare of the dog, safety of the veterinary staff, and the satisfaction of the owner.

The aim of this study was to assess the efficacy of a standardized desensitization and counter-conditioning training program on behavioural and physiological signs of fear during a veterinary visit. While tailored programs are likely to be most effective for treatment of individual dogs in this context, this approach requires careful planning and oversight by a professional with in depth behavioural knowledge. Instead, a standardized approach was used to replicate dog owners following set advice with minimal oversight, as provided by a primary care veterinarian during routine care. We hypothesized that the four-week training program would reduce fear levels in dogs during a veterinary visit with the prediction that training would reduce behavioural and physiological indicators of fear during arrival at the clinic and during a routine physical examination.

## 2. Materials and Methods

Owned companion dogs (n = 56; Table 1; only dogs included in the final analysis are counted and listed here) were recruited from local dog owners for participation. Dog owners were recruited online via Facebook posts on our laboratory Facebook page, the Ontario Veterinary College (OVC) page, on animal-related pages for the Guelph area, and via an internal electronic newsletter that was distributed to OVC staff and students. Paper advertisements were posted in-clinic at the Smith Lane Animal Hospital and on local community boards. To be selected for participation, dogs were required to be healthy, up to date on vaccinations, and ≥ 1 year of age. Dogs were eligible to participate if owners reported in the preliminary recruitment questionnaire that their dog displays mild to moderate fear (score of 2 or 3 on a 5-point scale ranging from 0 to 4) during an examination at the veterinary clinic. Only dogs with pre-existing mild to moderate fear were included in our study, as they were believed to be ideal candidates for our training program. Mild to moderate fear levels were chosen, as these dogs had room for improvement but were not above their threshold fear level. Dogs with severe fear (score of 4) would have required a tailored and carefully monitored training program in order to keep the dog and the owner safe and to result in noticeable improvement in their fear levels. Dogs were not eligible to participate if they had previously shown aggressive behaviour within the veterinary clinic in order to reduce possible bite risk to the investigators.

The study was conducted within a functioning veterinary clinic, the Smith Lane Animal Hospital at the Ontario Veterinary College, University of Guelph. The dogs were randomly allocated to treatment groups in a randomized block design, with sex and age balanced between treatment groups. Treatment groups consisted of training (n = 33) or no training (n = 23). Training involved instructing dog owners to desensitize and counter-condition their dogs to exam-style handling and the clinic environment through regular exposure across a four-week period. Assessment of each dog was conducted twice, before and after training, and these assessments occurred approximately four weeks apart [mean days apart (SD) was 29.8 (3.4) days]. During each test visit, dogs completed three stages of testing: arrival at the clinic, an acclimation period, and a standardized examination. All testing was conducted by the same two experimenters (examiner, Anastasia C. Stellato (ACS); handling assistant, Sarah Jajou (SJ)); both were female and wore white lab coats throughout testing. The duration of testing was approximately 20 min, and all testing was video recorded for later analysis. This research was approved by the University of Guelph Animal Care Committee and conformed to all federal and provincial guidelines governing use of animals in research (AUP #3907).

### 2.1. Stages of Testing

**Figure 1:**
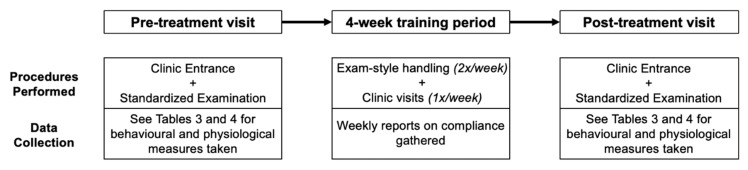
Stages of testing involving pre-treatment visit, four-week training period, and post-treatment visit. Dog owners not allocated to perform training were not given any instructions in between testing and thus were not emailed weekly.

For a detailed description of the stages of testing and the type of data collected at each stage, see Figure 1. The handling assistant greeted owners and test dogs outside the front doors of the veterinary clinic. The assistant video recorded the dog walking through two sets of entrance doors, through the waiting room, and stepping onto a scale in the front area to be weighed. In the waiting room, the assistant explained the testing protocol, and the owner was asked to read and sign the consent form. During this process, the assistant interacted with the test dog by speaking softly, offering treats, and petting when appropriate (i.e., dog approached the assistant and was not showing any obvious signs of fear or avoidance). This process took approximately 5 min. The owner then remained in the waiting area while the dog was taken to an examination room (3.56 m by 2.97 m). Although owners commonly accompany their dogs during routine veterinary examinations, for the current study, dogs were separated from their owners to reduce the potential impacts of owner behaviour on dog responses.

The dog was given one minute to acclimate to the room while the investigator and the handler stood still and off to the side without interacting with the dog. Each dog then completed a 7-min standardized physical examination (adapted from [8]), which started once all of the dog’s feet were on a non-slip rubber mat (0.9 m by 1.2 m; Rubber Master Mat). The investigator performed the examination in a pre-determined and standardized order. She examined the dog’s eyes, ears, and mouth (head exam), palpated the mandibular and the pre-scapular lymph nodes as well as the abdomen before moving hands over the dog’s body to check for abnormal masses and body condition (body palpation), and manipulated all four limbs (three for the dog with three legs) by running hands down each limb and lifting each paw (limb manipulation). The physiological measures were then assessed, which included measuring axillary temperature by placing a thermometer (Flexible Tip Digital Thermometer, Life Brand™, Thermor Ltd., Ontario, Canada) under the dog’s front leg until the thermometer beeped as well as auscultating with a stethoscope (3M™ 200 Littmann^®^ Classic III™ Stethoscope, St. Paul, MN, USA) for 15 sec to assess heart rate, and observing thoracic movements for 15 sec to assess respiratory rate. Heart rate and respiratory rate measures were multiplied by four to estimate the rate per minute. If the test dog showed signs of discomfort and/or aggressive displays during the examination, e.g., growling or extreme avoidance, the examination was either modified or terminated. Two dogs were removed from the training treatment group during the initial examination due to a high number of escape attempts and extreme avoidance.

### 2.2. Desensitization and Counter-Conditioning Training

After the examination, the investigator returned the dog to the owner in the waiting room. If the dog was randomly allocated to treatment, the owner was provided with detailed instructions for conducting desensitization and counter-conditioning training over the following four weeks. The training program was developed by the researchers as a formalized approach to anecdotal recommendations by veterinarians that fearful dogs should come in to the clinic for regular “cookie visits” and that owners should practice handling and exams at home. The frequency of training and the number of weeks of the training program were selected to balance training efficacy with practicality in terms of both owner compliance and owner expectations for improvement within a reasonable period of time and effort. A selection of dog owners were informally polled to determine how often they would be willing to practice handling and travel to the clinic for visits to assist their dog with this type of program, and many indicated that they would only be able to do it once or twice a week. Previous research indicated that 1 to 2 training sessions per week were sufficient for facilitating learning in dogs [31,32], thus owners were asked to visit the clinic at least once per week and to complete exam-style handling at least twice per week.

Owners were instructed to practice exam-style handling on their dog for 5–10 min twice a week at a location in the home where the dog was most comfortable. This included gradually exposing their dog to handling of different body areas, beginning with least-invasive methods (e.g., resting a hand on their paw) and advancing to more invasive methods (e.g., massaging the paw, Table 2) at a rate that was tailored to the individual dog’s response. Owners were advised to only advance to the next level of handling when the dog was calm and not displaying signs of fear or discomfort (e.g., lowered posture, avoidance/escape attempts, freezing, vocalizing, yawning, lip licking). They were also instructed to visit the veterinary clinic, Smith Lane Animal Hospital, for a minimum of 5 min once per week. This involved traveling to the clinic using their normal mode of transportation, having someone at reception greet their dog, walking their dog onto the scale, and then entering and staying in the designated examination room for at least 3 min. Handling sessions and clinic visits were paired with food rewards that were reported by the owner to be highly palatable to the dog (e.g., their favourite treat). All owners were provided with written handouts of all training protocols and a Supplementary Video that demonstrated the suggested handling progressions using two owned dogs (Video S1). The video material displayed different ways to administer treats during training; they were to be given either immediately after each individual handling attempt or paired directly with the handling, depending on the dog’s level of comfort with the handling procedure. If dogs were randomly allocated to the control group, owners were not given any specific instructions to follow during the four weeks in between testing. However, control group participants were provided with all study materials for reducing dog fear during veterinary visits at the end of the study. At the end of the study, all dog owners were asked if they were clients of the Smith Lane Animal Hospital, and if, during the four weeks, their dog had visited their veterinary clinic for purposes other than the study, or had received other forms of training. Also, owners who participated in training were asked if their dog consumed treats during the training sessions and if they noticed an improvement in their dog’s fear levels across the training sessions.

To monitor the training process and verify its completion, every week, owners from the training group were contacted and asked to complete an online survey detailing the number of sessions completed and any issues that were encountered. To be included in analysis, owners must have completed the training requirements for at least three of the four weeks. Five dogs (four in the training group and one in the control group) were lost to follow-up, and 12 additional dogs from the training group were removed from analysis due to non-compliance with the training program. Thus, the final sample sizes for analysis were n = 15 for training treatment group and n = 22 for control group.

### 2.3. Data Collection

All testing was video recorded using two video cameras (Sony Handycam HDR-CX330, Sony, Tokyo, Japan); one camera was placed in front of the dog (1.96 m away from mat) and the other was raised and positioned pointing downward to capture a rear view of the dog. An ethogram adapted from a previous study [8] was used to code all behaviours (Table 3). An individual that was blind to both the study hypothesis and the assigned treatment groups performed behaviour scoring using Observer XT software (Noldus Information Technology Inc, Wageningen, The Netherlands). Continuous sampling was used to score the frequency of all behaviours, with the exception of the following behaviours, which were scored as presence/absence: head, ear, and tail position, trembling, willingness to step on the scale, and vocalization. Behaviours were scored during each phase of the clinic arrival (entrance, weigh-in) and the examination (head exam, body palpation, limb manipulation, temperature assessment, heart rate assessment, and respiratory rate assessment), except for trembling, which was live scored (by the investigators who were not blind to treatment or hypothesis) across the entire exam to ensure accuracy. Scores for head, ear, and tail position were combined for analysis where a score of “absent” was assigned if 0 or 1 body part was reduced, and a score of “present” was assigned if 2 or 3 body parts were reduced.

In addition, dogs were subjectively scored from video for overall fear during clinic entrance and the examination using the same scoring system used by owners during preliminary recruitment (5-point scale ranging from 0 to 4; Table 4). These scores were used to ensure dogs met the minimum study inclusion criterion for mild to moderate fear and to assess changes in fear scores pre- and post-treatment. Scoring was conducted by one of the investigators (ACS) from videos and was de-identified and randomized, and scoring occurred more than 6 months after completion of participant testing; thus, there was the potential for the investigator to remember treatment assignment for some individual dogs, but they were blind to pre- versus post-treatment status.

### 2.4. Statistical Analysis

For behavioural measures, separate models were conducted for clinic arrival and examination. Generalized mixed models were used to assess the effects of training, testing phase (clinic arrival: entrance, weigh-in; examination: head exam, body palpation, limb manipulation, and temperature, heart rate, and respiratory rate assessment), sex, and age, with the dog as a random effect. The corresponding baseline behaviour performed during the first visit was included as a covariate in the analysis. Main effects in each model were identified using manual backward selection, with variables retained with a *p*-value < 0.05. After main effects were finalized, all possible interactions were tested, and any main effect involved in a significant interaction was retained in the model. Model fit was assessed, and residuals were evaluated to identify any outliers. For the clinic entrance, lip licking, encouragement needed to step on the scale, and avoidance were assessed with Poisson distributions, with duration of each phase (entrance and weigh-in) included as the offset. For posture, vocalizations, and willingness to step onto the scale, exact logistic regression was used. Testing phase was not assessed for willingness or encouragement, as a single measure was taken during the appropriate phase. For the examination, lip licking and avoidance were analyzed with Poisson distributions, with duration of each phase of examination included as the offset. For vocalizations, binary logistic distributions were used for analysis, and for trembling and posture, exact logistic regressions were used. Testing phase was not assessed for trembling, because it was a single measure taken across the entire examination. Body shaking and yawning were not analyzed due to the small number of dogs performing these behaviours.

For physiological measures taken during the examination (i.e., temperature, heart rate, respiratory rate), testing phase was not included in the models, since only a single measure was taken during the appropriate examination phase. The corresponding baseline measure taken during the first examination was included as a covariate. General linear models were used to assess the effects of training, age, and sex on these measures using the same model building strategies and techniques as above. Also, Wilcoxon signed rank tests were used to assess changes in fear scores from the first visit to the second, and Mann–Whitney tests were used to assess differences between treatment groups during first and second visits. All statistical analyses were performed with SAS v.9.3 (SAS Institute, Cary NC, USA). Data were deposited into Scholars Portal Dataverse (https://doi.org/10.5683/SP2/W8YG3G).

## 3. Results

From the sample of dogs selected for participation, 23 were male, 14 were female, five were tail docked, one had cropped ears (potentially affecting tail and ear position observations), one dog had three legs, and two dogs were sexually intact (one male and one female). A variety of breeds were included (23 mixed breeds, 14 purebreds), and the mean weight (SD) was 22.9 kg (10.78) and ranged from 3.2 to 42.9 kg. The dogs’ ages ranged from 1 to 13.5 years old. Also, nine dogs were patients of the Smith Lane Animal Hospital, and 28 were patients elsewhere. All dogs who were included in the study were scored by the investigator as displaying mild to moderate fear (a score of 2 or 3 on a scale of 0–4) during their first examination, with a median fear score of 2.

During the four weeks, nine dogs (two trained, seven control) visited their personal veterinary clinic for various procedures (e.g., routine examine, nail trim, vaccines, weigh-in), and three dogs (one trained, two control) received other forms of training (e.g., obedience, agility). Dogs who had certain procedures in between testing were not detected as outliers and could not be controlled for statistically due to limited degrees of freedom.

In regard to compliance with training protocols, more dog owners performed exam-style handling than clinic visits (Table 3). Out of the 27 dog owners responsible for training that completed the study, 12 (44%) were non-compliant (Table 5). The probability of compliant dog owners reporting an observable improvement in their dog’s fear levels across the training sessions was 86.7% (*p* = 0.007).

### 3.1. Clinic Entrance

#### 3.1.1. The First Visit

No behavioural differences were detected between treatment groups during the pre-treatment visit (*p* > 0.1), nor were any interactions detected between pre- and post-treatment responses. Performance of certain behaviours during the pre-treatment visit (baseline) influenced performance during the post-treatment visit. Specifically, demonstrated willingness to step on the scale (*p* = 0.008), encouragement needed to step on the scale (F_1,34_ = 4.18, *p* = 0.005), posture reductions (*p* = 0.0001), and avoidance (F_1,71_ = 4.49, *p* = 0.04) during the first visit increased the likelihood of these behaviours being performed during the post-treatment visit.

#### 3.1.2. Efficacy of Training during the Second Visit

**Figure 2:**
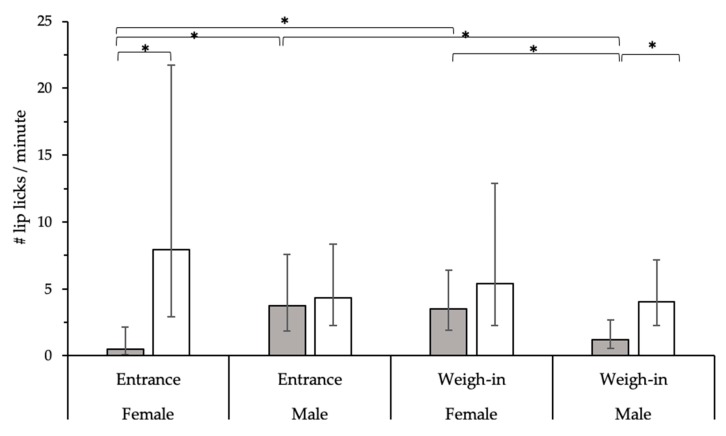
Differences in the rate of lip licking during post-treatment visit for control (gray) and trained (white) dogs varying in dog sex and phase of clinic entrance. A higher rate of lip licks (95% CI) was displayed in trained (white) females compared to control (gray) females during entrance and in trained males compared to control males during weigh-in; (mixed Poisson regression, * *p* < 0.05).

During clinic arrival, training influenced the rate of lip licking through an interaction with sex and phase (F_1,32_ = 11.09, *p* = 0.002; Figure 2). During entrance, trained females had a higher lip lick rate compared to control females, and during weigh-in, trained males had a higher rate compared to control males. Also, during entrance, control males had a higher rate of lip licking compared to control females, and during weigh-in, control females had a higher rate compared to control males. The performance of posture reductions (*p* = 1.00), vocalizations (*p* = 0.63), avoidance (*p* = 0.34), willingness to step on the scale (*p* = 0.19), or encouragement needed to step on the scale (*p* = 0.64) did not significantly differ across treatment groups.

#### 3.1.3. Subjective Fear Scores

For clinic entrance, fear scores did not differ between treatment groups during pre-treatment (*p* < 0.1) or post-treatment assessments (*p* > 0.1), and fear scores did not differ between pre- and post-training assessments for control (*p* > 0.1) and trained dogs (*p* > 0.1); for the post-training assessment, eight dogs had lower scores (three trained, five control), five dogs had higher scores (two trained, three control), and scores for 24 dogs remained unchanged (10 trained, 14 control).

### 3.2. Examination

#### 3.2.1. The First Visit

No behavioural or physiological differences between treatment groups were detected during the pre-treatment examination (*p* > 0.1), nor were any significant interactions detected in pre- and post-treatment responses. Trembling (*p* = 0.049), posture reductions (*p* < 0.0001), avoidance (F_1,176_ = 39.59, *p* < 0.0001), vocalizations (F_1,181_ = 25.21, *p* < 0.0001), lip licking (F_1,175_ = 4.49, *p* = 0.04), temperature (F_1,31_ = 5.17, *p* = 0.03), heart rate (F_1,31_ = 7.08, *p* = 0.012), and respiratory rate (F_1,33_ = 19.29, *p* = 0.0001) during pre-treatment exam (baseline) predicted the performance of these behaviours during the post-treatment exam.

#### 3.2.2. Efficacy of Training during Second Visit

**Figure 3:**
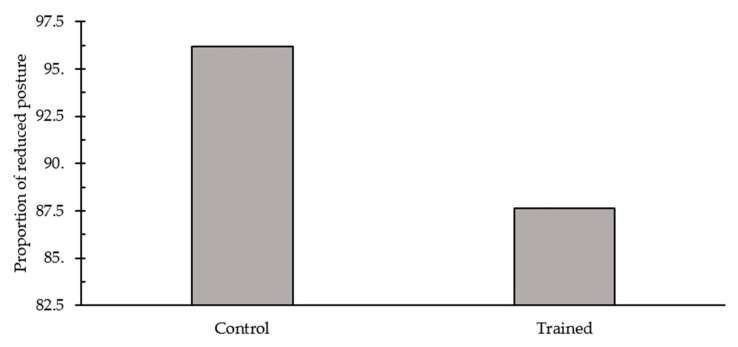
A higher proportion of control dogs (96.2%) displayed reduced posture during the examination compared to trained dogs (87.6%).

**Figure 4:**
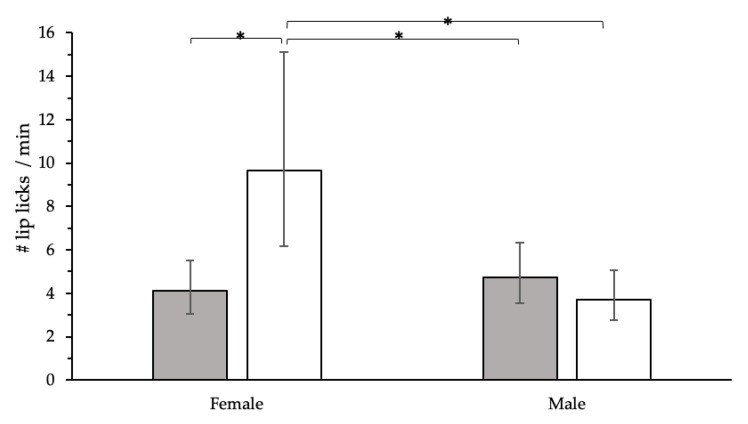
Interaction between training and dog sex from a mixed Poisson regression model displaying differences in lip lick rate (95% CI) during the post-treatment examination between control (gray) and training (white) treatment groups; * *p* < 0.05.

The training program influenced the performance of posture reductions (*p* = 0.03) during the post-treatment examination (Figure 3). There were higher odds (95% CI) of having reduced posture for control dogs (OR: 3.79, CI: 1.03–16.3) compared to trained dogs. During the examination, the training program influenced the rate of lip licking through an interaction with sex (F_1,33_ = 10.89, *p* = 0.002; Figure 4). Trained, female dogs had a higher rate of lip licks compared to control females and males. The performance of trembling (*p* = 1.00), avoidance (*p* = 0.43), and vocalizations (*p* = 0.30) did not significantly differ across treatment groups during post-treatment examination. Similarly, the physiological measures, temperature (*p* = 0.50), heart rate (*p* = 0.66), and respiratory rate (*p* = 0.07), did not significantly differ across treatment groups.

#### 3.2.3. Phase of Exam

The phase of the examination influenced avoidance behaviour (F_5,176_ = 4.8, *p* = 0.0004) and lip licking (F_5,175_ = 4.6, *p* = 0.0006). Avoidance was higher during head exam, limb manipulation, temperature assessment, and heart rate assessment compared to body palpation, and lip licking occurred at higher rates during the first three phases of the exam (head exam, body palpation, limb manipulation) in comparison to phases involving measurement of physiological variables.

#### 3.2.4. Subjective Fear Scores

Fear scores did not differ between treatment groups during the pre-training examination (*p* = 0.74) but did differ between treatment groups during the post-training examination (*p* = 0.046), with trained dogs having a median fear score of 2 compared to the score of 3 for control dogs. Fear scores did not differ between pre- and post-training examinations for control (*p* = 0.63) and trained dogs (*p* = 0.13); for the post-training assessment, five dogs had lower scores (four trained, one control), three dogs had higher scores (zero trained, three control), and scores for 29 dogs remained unchanged (11 trained, 18 control).

### 3.3. Age and Sex Effects

Dog sex influenced temperature measures through an interaction with baseline temperature (F_1,31_ = 7.51, *p* = 0.01). Female dogs had a higher initial temperature than males and had a greater reduction in temperature across time than males. Dog sex or age did not significantly influence the effectiveness of the training program.

## 4. Discussion

### 4.1. Effect of Desensitization and Counter-Conditioning Training

We predicted that dogs exposed to a standardized four-week program of desensitization and counter-conditioning training would display fewer behavioural and physiological measures of fear during their post-treatment visit to the veterinary clinic compared to control dogs. The performance of posture reductions was reduced for dogs exposed to the training program, with trained dogs having lower odds of reduced posture compared to control dogs during the post-treatment exam. Reduced posture is a widely used indicator of fear in dogs and has been previously observed in response to a variety of stressors including physical restraint [33], physical examination [2,8,11], novel and startling stimuli, e.g., opening umbrella [34], noise [35], social isolation and restricted housing [36], kennelling in a veterinary clinic [37], and positive punishment [38]. Also, subjective researcher-completed fear scores were lower during the post-treatment examination. However, as a major component of the fear score was dog posture, the fear scores are potentially correlated with the performance of posture reductions. In addition, owners perceived an observable improvement in their dogs’ fear levels across the training period. Thus, despite no other behavioural or physiological effects detected, results suggest the training program was mildly effective at reducing fear levels in dogs.

In contrast to our predictions, post-treatment levels of lip licking were higher for dogs in the training group compared to dogs in the control group. Lip licking has been discussed as an appeasement signal displayed by dogs in response to a threatening stranger [39], to handling [27,28], as well as a sign of acute stress in response to unpredictable stimuli within a social setting [33], and to an examination [8,11,40]. Thus, the current results might indicate that fear levels were higher in the dogs in the training group. However, it is more likely that dogs in the current study were anticipating food delivery since their training visits had been paired with food rewards. Also, as lip licking has been associated with social interactions [33,39], it is possible that additional interactions with people in the clinic resulted in an increased expression of lip licking during the post-training assessment. Also, there was an influence of dog sex, as during the entrance stage trained, female dogs displayed more lip licks than controls, and during weigh-in, trained male dogs displayed more lip licks. However, during the exam, this effect was only present in female dogs, thus this requires further exploration to fully understand how dog sex plays a role in this context. Therefore, the results for lip licking should be interpreted with caution, and more research is necessary to explore these relationships and to assess these hypotheses.

The approximate frequency and duration of training used in the current program have been effective in other contexts and species. For instance, previous studies have demonstrated that dogs who received one training session one to two times per week had better acquisition of learned tasks compared to dogs trained more frequently [31,32]. Similar findings have also been detected in rats [41,42] and ponies [43]. Moreover, Demant et al. [31] reported that, after four weeks of training, memory retention was not influenced by training frequency. Also, previous research suggests that four training sessions involving desensitization and counter-conditioning dogs to oral gavage reduced behavioural indicators of poor welfare (e.g., reduced posture, escape attempts) [44]. Although the training program only influenced one fear response in the predicted direction, dog owners reported reduced fears signs in their dogs throughout the four-week training period. In addition, all testing took place in a functioning veterinary clinic, and therefore the contributing stimuli, e.g., smells, sounds, people in the waiting room, were incorporated within the training program, enhancing the external validity of our results. Despite previous studies support for the general approach of our training regimen, it is possible that improved results might be achieved with a more intensive or longer duration training program.

The training program was designed to be a practical, owner-completed program that could be implemented independently based on veterinarian recommendations and guidelines. While this program was designed to be practical and achievable for owners, compliance was relatively poor. Similarly, owner compliance in following training recommendations was an issue when owners were asked to perform desensitization and counter-conditioning techniques to thunderstorm phobic dogs [24]. This low compliance occurred even with a sample that was recruited in part from the OVC community and was likely therefore skewed towards more motivated owners. In real-life situations, compliance is likely to be even lower, since owners in the current study were dedicated to contributing to the research and were also provided with a monetary incentive. As the owners voluntarily participated in our study, it is likely that they represent a sub-sample of responsible and committed dog owners, and previous research suggests that even highly responsible dog owners are not compliant to conducting training and socializing due to perceived difficulty or lack of time [45]. Future studies should therefore examine the effects of having the training conducted by a professional trainer to determine whether this increases compliance with the training program.

Although the training program was only effective at reducing posture reductions, owners who participated in training reported a noticeable improvement in their dog’s fear levels across the training period. This owner-reported improvement is beneficial because if owners believe their dog is improving and becoming less fearful when being handled or visiting a veterinary clinic, it may motivate them to increase attendance at their veterinary clinic. Also, recent research suggests that dogs are sensitive to handler emotional states and potential emotional contagion, thus having a positively valanced (e.g., motivated and satisfied) owner may act to reduce dog fear behaviours, such as vocalizing, shaking, or panting [46]. Owners have been found to be fairly reliable in identifying general fear in dogs [47], and it is possible that the subjective owner reports captured an aspect of dog behaviour that was not assessed in the more formal analysis. However, a placebo effect is also possible, and owner reports for control dogs were not assessed for comparison. Therefore, as this is a subjective and potentially biased measure based on reports from compliant and motivated owners, this result should be interpreted with caution. Further research should explore and compare owner and investigator assessments of fear behaviours post-training.

### 4.2. Age and Sex Effects

During the examination, temperature showed a greater reduction from the first visit to the second for both trained and control females compared to males. It has been demonstrated that, when an animal is under stress, their body temperature increases in response [5,48], suggesting that females may have been at a higher level of fear during their initial visit and had reduced fear levels during their final visit. These findings remain consistent with other studies that have reported female dogs to be more fearful than males during examinations [2,11]. Also, it is possible that being exposed to the standardized, low-stress examination during the first visit caused their second exposure to be perceived as less aversive through some level of habituation for both groups. Others have suggested that repeated exposure to handling can result in reduced stress due to its predictability [49]. However, further research is necessary to fully explore this relationship, possibly through a more intensive training program.

### 4.3. Phase Effects

Similar to other studies, [8,11], fear behaviours (e.g., avoidance and lip licking) were influenced by the phase of the examination, with most fear behaviours occurring during the more invasive physical manipulations compared to the less invasive physiological assessments of temperature, heart rate, and respiratory rate.

### 4.4. Limitations

Handling in the current study was completed by owners in the home, and it is possible that these experiences might not have translated to unfamiliar handlers within the veterinary context without the owner present. Despite the owners performing clinic visits to counter-condition the dogs to veterinary stimuli, the handling was performed separate from these visits, and a greater treatment effect might have been observed with the owner present in the room. Although owners generally accompany their dogs during examinations, it was important to remove any impacts owners might have had on their dog’s responses. Also, as dogs are often separated from their owner during routine veterinary care, the responses observed during separation are still valid. To address these concerns in future research, owners should be invited in the examination room during testing and/or training sessions should be completed by the investigator in the veterinary clinic or the participant’s home. The latter might also be helpful for improving compliance. In addition, veterinary-related experiences that occurred outside of the training program during the four-week training period might have influenced dog responses. For example, dogs who had a negative experience during the treatment period might have been less responsive to treatment or even had an increase in veterinary-related fear as a result of that experience. However, only two of the nine dogs who had visited their veterinary clinic during the study period were within the training treatment, thus it is unlikely that our data were affected by these experiences. Also, by advertising our study to patients who visited the Smith Lane Animal Hospital, some of the dogs had prior exposure to the clinic that was used for testing. More research is necessary to understand how other experiences around the visit influence responses and to examine whether certain dog or fear characteristics influence responsiveness to training.

Further, beyond owners reporting compliance with the training program, it was not possible to assess the quality of the training completed by the owner. It is possible that owners went too quickly, resulting in flooding, which might sensitize the dog to the clinic environment and exam-style handling. For instance, previous research by Hall et al. [44] found that sham dosing dogs prior to dosing without proper training resulted in sensitization to the event, as shown in their increased performance of escape attempts and reduced posture. However, no dogs with extreme fear were enrolled in the study, and the current data do not suggest that dogs became more fearful during training; therefore, the program, although ineffective, likely did not become a flooding program. If any overt fear behaviours were detected during the training session, we provided detailed instructions for owners to terminate the session and to begin the next session with less invasive methods, with advancements made based on their dog’s observed behaviour. Also, although owners reported improvement in their dog’s fear levels, owners did not report their progress with the different stages of the handling progressions, thus we do not know if they were able to complete all stages of handling by the end of the program. To avoid this in future studies, the endpoint could be based on completion of the full program rather than an arbitrary set schedule.

It has been suggested that food rewards might not be the most effective reinforcer for desensitization and counter-conditioning training in this type of context, since recent studies show that dogs are less likely to eat a treat inside the clinic than outside [10]. However, owners were instructed to keep their dogs below their fear threshold during training and were encouraged to use high value treats. The majority of dog owners reported their dogs generally consumed treats while in the veterinary clinic as well as during the training sessions, indicating that food rewards were effective as a reinforcer for this study. For the few dogs who were less likely to consume treats when under stress, the training was less likely to be effective; playing may have been useful as a reinforcer for these dogs, as it has been a successful reinforcer for young rats [50], and Affenzeller et al. [51] discovered that playing after a period of training reinforced learning for dogs. However, further research is necessary to explore the efficacy of these practices in desensitization and counter-conditioning training.

## 5. Conclusions

The current standardized four-week training program improved dog posture levels and influenced owner satisfaction with their dog’s fear levels but did not influence any other indicators of fear, suggesting that the program was only mildly effective. Thus, owners and veterinarians should be aware that the benefits of this type of short-term, owner-based training program are somewhat limited. Veterinarians should also be aware that client compliance with this type of program is relatively low, even with provision of detailed instructions. Further research should therefore explore the effects of longer and more intensive training programs with higher intensity training sessions, potentially with tailoring to each individual dog’s progress and individual characteristics. Also, future studies should explore the effects of a veterinarian- or trainer-completed training program, where the passive exam-style handling is completed on the dogs on a weekly basis at the veterinary clinic.

## Figures and Tables

**Table 1:** Characteristics of dog participants (n = 37).

Dog	Breed	Age (Years)	Sex	Training/Control
Avery	Yorkshire Terrier	3	F	Training
Bear	Mixed Breed	3	M	Training
Bugsly	Mixed Breed	9	M	Training
Dobby	Mixed Breed	3	M	Training
Finn	Mixed Breed	2	M	Training
Ivy	Mixed Breed	2	F	Training
Midori	Mixed Breed	5	F	Training
Monty	Greyhound	7	M	Training
Prince	Mixed Breed	9	M	Training
Rose	Mixed Breed	3	F	Training
Solo	Mixed Breed	1	M	Training
Tanner	Retriever (Golden)	3	M	Training
Tucker	Other	6	M	Training
Waldo	Mixed Breed	7	M	Training
Zappa	Miniature Pinscher	11	M	Training
Addison	Collie (Rough)	7	F	Control
Basil	Beagle	2	M	Control
Calista	Mixed Breed	2	F	Control
Carter	Mixed Breed	3	M	Control
Daisy	Mixed Breed	4	F	Control
Duncan	Mixed Breed	7	M	Control
Dusty	Mixed Breed	8	M	Control
Essie	Mixed Breed	1	F	Control
Freya	Bulldog	1	F	Control
Hunter	Spaniel (English Springer)	7	M	Control
Kalaylee	Havanese	3	F	Control
Lucy	Mixed Breed	2	F	Control
Luis	Dachshund	13.5	M	Control
Maggie	Other	3	F	Control
Milo	Mixed Breed	9	M	Control
Oliver	Mixed Breed	2	M	Control
Penny	Labradoodle	6	F	Control
Pepper	Mixed Breed	11	F	Control
Reggie	Mixed Breed	6	M	Control
Roo	Mixed Breed	3	F	Control
Wrangler	German Shepherd Dog	7	M	Control
Zeus	Mixed Breed	1	M	Control

**Table 2:** Suggested handling progressions for owners to use during training, with the instruction to begin with the body part the dog is most comfortable with and to only proceed to a more advanced progression when the dog is calm.

Body Part	Progressions
Paws	Place hand beside their paw on the ground Touch their paw (one at a time, for short intervals of time; 2–3 sec) Hold their paw (for short intervals of time; 2–3 sec) Hold their paw progressively for longer periods (10–15 sec) Massage their paw
Mouth	Place your hand below their ear Stroke the sides of their muzzle Touch their lips (one side at a time) Gently lift the lip exposing their teeth (for short intervals of time; 2–3 sec) Progressively expose their teeth for longer periods (10–15 sec)
Ears	Touch the back of their head Touch their ears (one at a time) Rub their ears Pull back the ear to expose the inside (for short intervals of time; 2–3 sec) Progressively expose their ear for longer periods (10–15 sec)
Body	Pet them along the top of their back, from nape of the neck to before hips Move hands onto their sides and chest, petting from shoulder to before hips Move hands around their body in massage-like circular motion (including chest, sides, and back, and belly)

**Table 3:** Ethogram of behaviours scored during all stages of the veterinary visit.

Testing Phase	Behaviours	Description
All phases	Head position	
	i. Neutral	Head neutral or high
	ii. Reduced	Head low
	Tail position	
	i. Neutral	Tail high or breed specific position
	ii. Reduced	Tail lowered either still or wagging, or tucked between bent hind legs
	iii. Dog tail out of view	Cannot determine tail position
	Ear position	
	i. Neutral	Ears forward
	ii. Reduced	Ears sideways, down, or pinned back
	iii. Dog ears out of view	Cannot determine ear position
	Other behaviours	
	Body shaking	Lateral, side to side rotation of the body about the central axis, with shaking of the fur
	Lip/Snout licking	Portion of the tongue moves along the upper lip
	Yawning	Wide opening of mouth
	Vocalizing	Barking, growling, whining, yelping
Clinic Entrance	Avoidance	Moving/manipulating body away, refusing to move forward, or successfully placing at least one paw off the scale
	Encouragement to step on scale	Number of times the owner needed to encourage them onto the scale
	Willingness to step on scale	Forced on by pulling on leash, or physically moving them
Examination	Trembling	Obvious shivering of the body
	Avoidance	Moving/manipulating head or body away from the investigator or handling device
	Escape	All four paws off the mat

**Table 4:** Method for subjective scoring of dog fear on a 5-point scale ranging from 0 (no fear) to 4 (extreme fear). This scale was used by participants during preliminary recruitment and by the investigator for assessing overall fear scores during clinic entrance and examination.

Score	Behaviour Description	
0	*Posture:* Head normal or high, ears forward, tail high or breed specific position *Avoidance:* No escape attempts *Subtle behaviours ^a^:* None	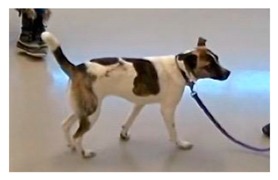
1	*Posture:* Head normal or high, ears forward or slightly back from neutral, tail body-height or slightly lowered *Avoidance:* No escape attempts *Subtle behaviours ^a^:* 1-2 displayed	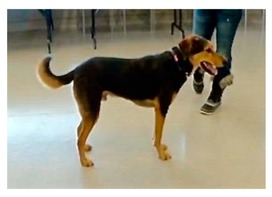
2	*Posture:* Head normal or low, ears sideways, tail lowered and either still or wagging *Avoidance:* Minor attempts to escape, retreat or hide; few steps made backwards/away *Subtle behaviours ^a^:* Several behaviours displayed	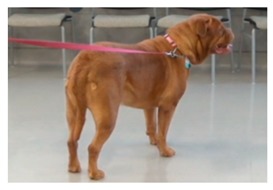
3	*Posture:* Head somewhat low, ears down or slightly back, tail tucked between legs *Avoidance:* Moderate attempts to escape, retreat or hide; more than a few steps made backwards/away *Subtle behaviours ^a^:* Several behaviours displayed	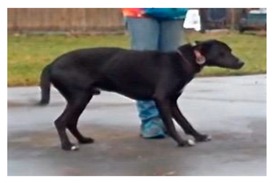
4	*Posture:* Head exaggeratedly low, ears down and pinned back, tail fully tucked between bent hind legs *Avoidance:* Vigorous/constant attempts to escape, retreat or hide *Subtle behaviours ^a^:* Several behaviours displayed	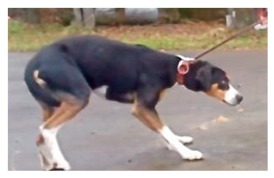

^a^ Subtle behaviours include: yawning, lip licking, body shaking, vocalizing, trembling.

**Table 5:** Owner reported compliance with training protocols for all dog owners in the training treatment group (n = 27) displaying the number of owners who performed exam-style handling on their dog and brought their dog to the clinic for the required minimum number of sessions over the course of the study. Compliant owners (n = 15) completed three or four weeks of training, and non-compliant owners (n = 12) conducted training for zero, one, or two weeks.

		Exam-Style Handling		Total
		Yes	No	
Clinic Visits	Yes	15	0	15
	No	4	8	12
Total		19	8	27

## Supplementary Materials

The following are available online at https://vimeo.com/276290339/9d5d0f6a3d, Video S1: Handling techniques owners were instructed to perform during handling sessions with their dog.
