# Supplementary material for: Effect of a Standardized Four-Week Desensitization and Counter-Conditioning Training Program on Pre-Existing Veterinary Fear in Companion Dogs
Source: Animals (Basel). 2019 Oct 7;9(10):767. doi: 10.3390/ani9100767 (PMC6826973; doi:10.3390/ani9100767)
Supplement: Supplementary file 1 [file animals-09-00767-s001.zip › Supplementary Materials/Video Link.docx]

**Handling Progression Supplementary Video**

Please use the following private link to access a video that demonstrates the various handling techniques owners were instructed to perform during handling sessions with their dog:

<https://vimeo.com/276290339/9d5d0f6a3d>
