# Supplementary material for: Effect of a Standardized Four-Week Desensitization and Counter-Conditioning Training Program on Pre-Existing Veterinary Fear in Companion Dogs
Source: Animals (Basel). 2019 Oct 7;9(10):767. doi: 10.3390/ani9100767 (PMC6826973; doi:10.3390/ani9100767)
Supplement: Supplementary file 1 [file animals-09-00767-s001.zip › Supplementary Materials/Dataset.docx]

**Dataset**

Private URL:

<https://dataverse.scholarsportal.info/privateurl.xhtml?token=2ed4b8a2-2fd6-4069-a5a8-8d0d772ec3bc>

The data has also been deposited in a reputable repository and will be made publicly available upon manuscript acceptance. The dataset citation, including the registered DOI is:

Stellato, Anastasia C.; Jajou, Sarah; Dewey, Cate E.; Widowski, Tina M.; Niel, Lee, 2019, "Effect of a standardized 4-week desensitization and counter-conditioning training program on pre-existing veterinary fear in companion dogs", <https://doi.org/10.5683/SP2/W8YG3G>, Scholars Portal Dataverse
